# Supplementary material for: Nutritive Value and Bioactivities of a Halophyte Edible Plant: Crithmum maritimum L. (Sea Fennel)
Source: Plants (Basel). 2024 Jan 31;13(3):427. doi: 10.3390/plants13030427 (PMC10857157; doi:10.3390/plants13030427)
Supplement: Supplementary file 1 [file plants-13-00427-s001.zip › plants-2798289-supplementary.pdf]

Supplementary material - Plants

Nutritive value and bioactivities of a halophyte edible plant: *Crithmum maritimum* L. (sea-fennel)

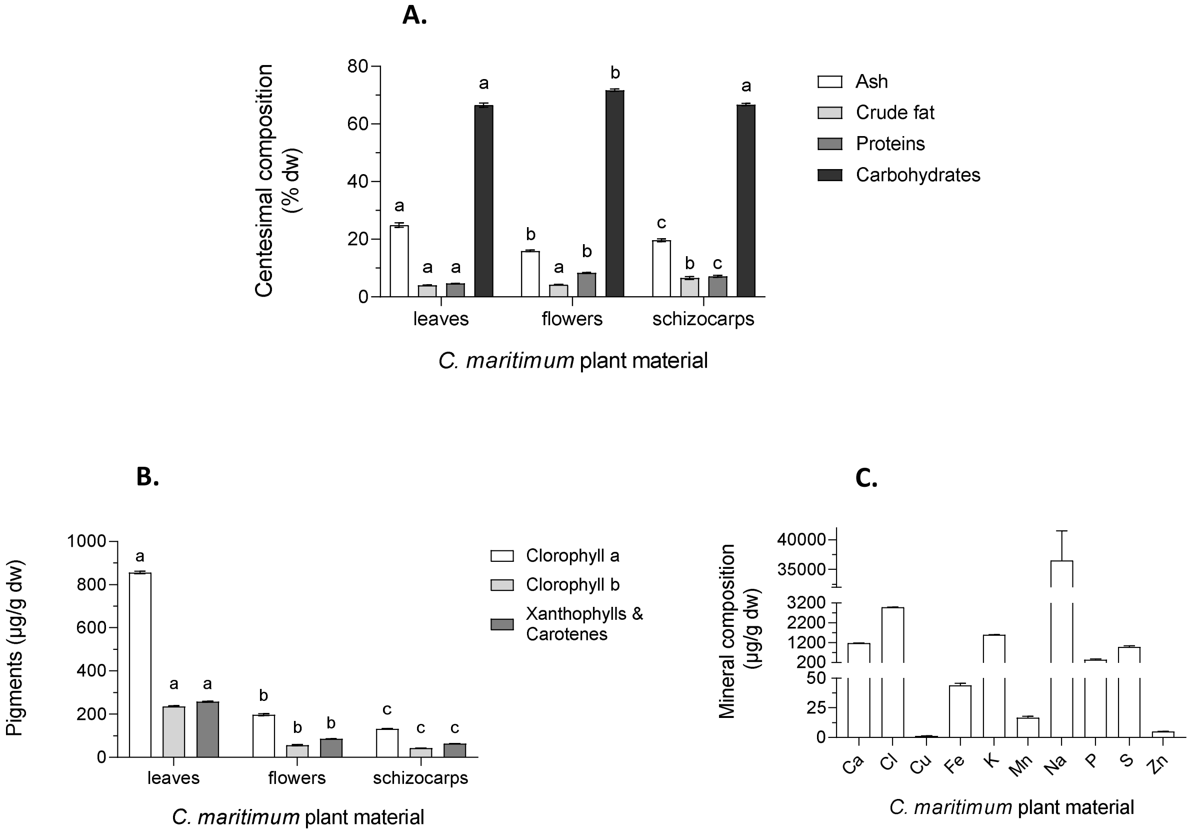

Figure S1. *C. maritimum* centesimal composition and contents in pigments and minerals. (A) Centesimal composition of sea fennel leaves, flowers, and fruits/schizocarps (in % dw). (B) Contents in photosynthetic pigments (Chlorophyll a and Chlorophyll b), xanthophylls and carotenes (in µg/g dw). (C) Mineral composition of whole aerial parts in Ca, Cl, Cu, Fe, K, Mn, Na, P, S and Zn (in µg/g dw). One-way ANOVAs were applied to analyse differences between the different types of plant material in (A) and (B). Different letters across the columns mean significant differences ($p < 0.05$).

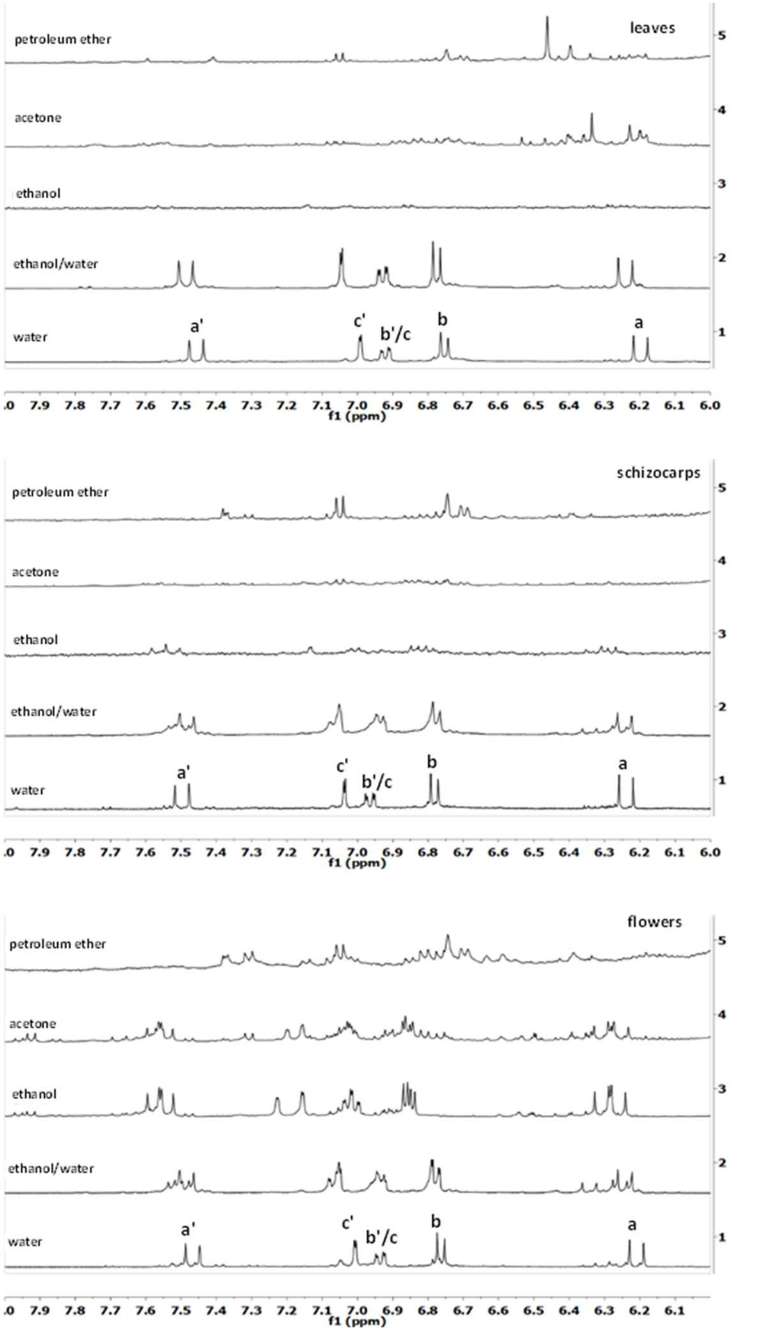

Figure S2. Downfield aromatic regions (δ 6.0 to 8.0 ppm) of 1D ^{1}H NMR spectra of extracts of leaves (A), fruits/schizocarps (B), and flowers (C) of *C. maritimum L.* (sea fennel) in petroleum ether, acetone, ethanol, 1:1 ethanol/water and water populated by doublet signals (*d*), assigned to meta- and ortho-coupled protons of phenolic moieties and vinyl groups of hydroxybenzoic and hydroxycinnamic acids: (chemical shift (δ), multiplicity, vicinal coupling constant (^{3}J)): (a') 7.50 (*d*, J = 15.9 Hz), (c') 7.00 (*d*, J = 2.0 Hz), (b' and c) 6.95 (*dd*, J = 8.4 Hz, 2.0 Hz), (b) 6.80 (*d*, J = 8.4 Hz), (a) 6.25 (*d*, J = 15.9 Hz).

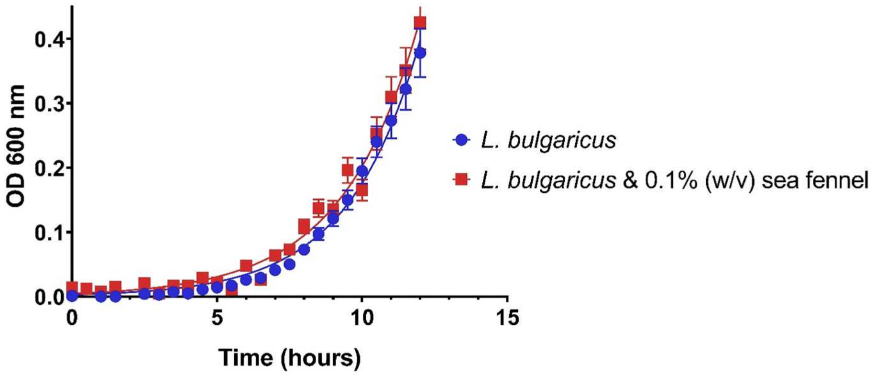

Figure S3. *Lactobacillus bulgaricus* growth curves. Bacterial growth monitored via optical density (OD) at 600 nm for 12 h, at 37 ºC, in the absence and presence of *C. maritimum* (sea fennel) leaves at 0.1 % (w/v).
